# When Workers Feel Like Objects: A Field Study on Self-Objectification and Affective Organizational Commitment

**DOI:** 10.5964/ejop.5549

**Published:** 2023-02-28

**Authors:** Roberta Rosa Valtorta, Maria Grazia Monaci

**Affiliations:** 1 Department of Psychology, University of Milano-Bicocca, Milan, Italy; 2 Department of Human and Social Science, University of Valle d’Aosta, Aosta, Italy; Glasgow Caledonian University, Glasgow, United Kingdom

**Keywords:** dehumanization, objectification, self-objectification, affective organizational commitment, workers

## Abstract

Objectification is a form of dehumanization that implies the perception of others as mere objects. The present study aimed to expand research on objectification in the work domain by exploring the relationships between objectifying job features, self-objectification, and affective organizational commitment within a real work setting. Building on previous literature, we hypothesized that the execution of objectifying work activities would be positively related to workers’ tendency to objectify themselves. Further, we expected a decrease in affective organizational commitment as the outcome of these perceptions. A study involving 142 Italian supermarket clerks (75 females) supported our hypotheses. Workers with a low-status job role (i.e., cashiers and salespeople vs. managers) perceived their activities as more objectifying. In turn, this perception heightened their self-objectification, which decreased workers’ commitment towards the organization. Our results enrich the understanding of workplace objectification by also providing relevant insights into the link between social-psychological and organizational processes.

Objectification is a form of dehumanization that involves the perception of others as objects, instruments, or goods (LaCroix & Pratto, 2015; Volpato & Andrighetto, 2015). In particular, the philosopher Martha Nussbaum (1995) specified seven ways to objectify a person, including treating others as instrumental, fungible, violable and owned as well as denying others autonomy, agency, and subjectivity. During the last decades, social psychology has generally focused on sexual objectification, that is, the experience of being treated as a body (or collection of body parts) valued predominantly for its use to (or consumption by) others’ (see Gervais, 2013; Loughnan & Pacilli, 2014, for reviews). However, objectification is a much broader phenomenon that may encompass many human interactions and domains. Perceiving others as mere objects is indeed a powerful cognitive strategy that rationalizes their exploitation or subordination (Volpato et al., 2017).

Marx (1844) claimed that workers in a capitalistic society are denied the traits that define their humanity and are judged exclusively for what they produce. In Marx’s view, the capitalist model’s goal is to produce wealth, and workers are the essential instruments in creating this wealth. Although these reflections may appear to belong to a past era, workers’ objectification still permeates many workplaces. A report (BBC, 2013) documented the object-like treatment of Amazon “order-pickers.” Their daily activity is highly repetitive, imposed by a timer, and mostly limited to picking orders from supervisors and finding products in the warehouse. More recently, a former worker at Amazon’s Swansea warehouse has claimed that clerks were treated like robots and routinely sacked for not meeting “unrealistic targets” (BBC, 2018). Similarly, a picker at a warehouse in Minnesota has stated that Amazon workers were considered more similar to machines than human beings (BBC, 2019).

Despite the relevance of these considerations to workers’ well-being, only recently social psychologists have begun to investigate objectification in the workplace. For example, Baldissarri et al. (2017a) showed that the activities performed by a factory worker led to an association of the work with alienation. In turn, work that is perceived as potentially alienating—something that can estrange the worker from him/herself—triggered a worker’s perception no longer as a human being but as an object. A growing amount of studies have added a tile to this picture by revealing that objectification in the workplace can be triggered by multiple factors (e.g., Baldissarri et al., 2017b; Wang & Krumhuber, 2017). For instance, Andrighetto et al. (2017) showed that workers performing subordinate activities characterized by repetitive movement, fragmented activities, and other direction were perceived as instrument-like and less able to experience human mental states. Consistent with these findings, Loughnan et al. (2017) found that merely recalling an objectifying work experience led employees to perceive themselves as less human. Furthermore, Valtorta et al. (2019a) found that, because of their characteristics, most subordinate job activities were associated with an objectifying image of workers.

Baldissarri and colleagues (2017b) provided evidence for the effect of objectifying job features and self-objectification. In particular, the authors found that performing a manual or a computer task that was repetitive, fragmented, and other-directed was a relevant antecedent of working self-objectification per se, which led people to objectify themselves more than when performing a corresponding but non-objectifying activity. Further, the authors found that this increased self-perception as object-like led, in turn, to a decrease of belief in having free will, that is, the perception of having the ability to make free and conscious choices (Baumeister & Monroe, 2014; Feldman, 2017). By expanding these results, Andrighetto et al. (2018) showed that working objectification and the consequent self-objectification could also lead to an increase in conforming behaviors. Furthermore, Rollero and Tartaglia (2013) focused on the consequences of objectification in the occupational domain and found that objectified individuals were considered more suitable for low-status job activities than non-objectified ones.

More recently, Caesens et al. (2014; see also Caesens et al., 2019) speculated that dehumanization in the work domain has a strong relationship with attachment and intentions to leave the organization. Indeed, several authors argued that perceptions and self-perceptions of humanity within a work setting might impact both employees’ subjective well-being and organizational outcomes (e.g., Bell & Khoury, 2016; Caesens et al., 2017; Christoff, 2014). In this sense, prior studies reported that dehumanizing perceptions are positively related to employees’ emotional exhaustion, psychosomatic strains, and turnover intentions, and negatively associated with workers’ job satisfaction (e.g., Baldissarri et al., 2014; Bell & Khoury, 2016; Caesens et al., 2017). Crucially, Bell and Khoury (2011) claimed that feelings of dehumanization should also be related to affective organizational commitment, namely the relative strength of an individual’s identification with and involvement in a particular organization (Mowday et al., 1982). Dehumanization is indeed a negative experience that diminishes the individual humanity and is thus likely to motivate the individual to dissociate from the organization. In addition, according to several scholars (e.g., Caesens et al., 2017; Christoff, 2014), dehumanizing perceptions and the internalization of these views might impair workers’ well-being by enhancing, for example, their level of anxiety or depression as it thwarts basic individual needs such as the needs for competence or affiliation.

Organizational commitment has been the subject of numerous investigations over the last few decades, and this is due mainly to its importance for employees and employers (see Yousef, 2017). Meyer and Allen (1991; see also Meyer et al., 2012) identified three forms of organizational commitment: affective commitment, namely the emotional attachment to the organization, based on identification with collective goals; continuance commitment, which refers to the costs associated with leaving; normative commitment, which is a felt moral obligation to stay. Literature has shown that, compared to normative and continuance commitment, affective commitment is associated with more variables and more strongly with each of them (see, e.g., Meyer et al., 2002; Trifiletti et al., 2014). Indeed, affective organizational commitment tends to relate positively to employees’ well-being (Meyer & Maltin, 2010) and negatively to psychosomatic symptoms (Addae & Wang, 2006; Richardsen et al., 2006). Despite these relevant findings, no previous research has investigated affective commitment by analyzing its link with dehumanization and objectification in the work domain. Therefore, through the present field study, we aimed to fill this gap in the literature by deeper examining working objectification in the supermarket sector and its potential antecedents and consequences, especially in terms of objectifying job features and affective commitment towards the organization.

Considering the findings and suggestions reported above, we first assumed that performing a repetitive, fragmented, and other-directed work activity (i.e., objectifying job features) would be associated with workers’ tendency to objectify themselves (*Hypothesis 1*). Furthermore, we assumed that this association would emerge especially among workers with a low-status job role. Importantly, we explored the link of these perceptions with affective organizational commitment, assuming that, in turn, self-objectification would be associated with a decrease in the workers’ commitment towards the organization (*Hypothesis 2*). We tested our assumptions among workers of supermarkets, where low-status employees (e.g., cashiers) typically perform objectifying work tasks. As reported by Baldissarri and colleagues (2014), this work context is indeed characterized by job features strongly related to the critical conditions of objectification identified in previous research (e.g., Blauner, 1964; Nussbaum, 1995), such as repetitiveness, fragmented tasks, and dependence on the machine.

## Method

### Participants

One hundred and forty-two workers (75 females) employed in full-service supermarkets in Piedmont, Italy, participated in the study in July 2019. The age distribution ranged between 18 and over 50, with approximately 60% of the respondents reported an age between 26 and 40 years old. Participants were employed in different job roles, such as salespeople (*n* = 81, 57%), cashiers (*n* = 50, 35%), and managers (*n* = 11, 8%) (for more details on demographic characteristics by job role, see **Table 1**). A sensitivity power analysis for a linear multiple regression with 3 predictors, using G*Power (Faul et al., 2009), indicated that the *f* ^2^ = .08 would be the minimum effect size that this sample can detect with 80% power (α = .05).

**Table 1:** Demographic Characteristics by Job Role

Characteristic	Total, *n* (%)	Salespeople, *n* (%)	Cashiers, *n* (%)	Managers, *n* (%)

Gender				
Male	67 (47%)	45 (56%)	15 (30%)	7 (64%)
Female	75 (53%)	36 (44%)	35 (70%)	4 (36%)
Age in years				
18-25	22 (15%)	10 (13%)	12 (24%)	0
26-30	32 (22%)	18 (22%)	14 (28%)	0
31-35	25 (18%)	17 (21%)	7 (14%)	1 (9%)
36-40	27 (19%)	17 (21%)	6 (12%)	4 (37%)
41-45	21 (15%)	13 (16%)	5 (10%)	3 (27%)
46-50	11 (8%)	5 (6%)	4 (8%)	2 (18%)
50 and older	4 (3%)	1 (1%)	2 (4%)	1 (9%)
Grand total	142	81	50	11

### Design and Procedure

With adult participants and anonymous questionnaires, ethical approval was not required, in line with national guidelines of the Italian Association of Psychology (AIP). All procedures performed in the study were in accordance with the APA ethical guidelines and the ethical principle of the “Helsinki Declaration” and the Oviedo Convention on Human Rights and Biomedicine. Full informed consent was obtained in writing before participants started the study.

We chose some Italian supermarkets on the basis of our connections. Then, we presented the study to the management and requested authorization to collect data among all the workers of the company. Supermarket clerks were recruited by word of mouth and fliers placed in the supermarkets with the assistance of the management and senior human resources specialists of the participating companies. Finally, one investigator administered individually to each worker a paper-and-pencil questionnaire presented as a survey on “attitudes and perceptions of workers.” Participants completed the survey at the supermarket where they work and during their break time. After completing the scales described below, participants were asked some demographics, were thanked and fully debriefed.

### Measures

#### Perception of Objectifying Job Features

The workers’ perception of their activity as objectifying was measured by using five items regarding repetitiveness, fragmentation, and other direction (e.g., “The job is quite simple and repetitive”; α = .78) already used and validated in previous research (e.g., Baldissarri et al., 2019; Hackman & Oldham, 1976). Participants were asked to rate the extent to which their job had these characteristics on a 5-point scale (1 = *not at all*, 5 = *extremely*). Higher scores denote greater workers’ perception of their activities as being characterized by objectifying job features.

#### Self-Objectification

To measure self-objectification, workers were asked to rate the extent to which they perceived themselves to be similar (1 = *not at all*, 5 = *extremely*) to four instrument-related words (i.e., *instrument*, *tool*, *thing*, *machine*; α = .81) and three human-related words (*human being*, *person*, *individual*; α = .66) during their work activity. These items were previously used in research concerning objectification in the work domain (e.g., Baldissarri et al., 2017a, 2017b, 2019). A single index was computed by subtracting the human-related score from the instrument-related score so that higher scores indicated greater self-perceptions as being instrument-like (vs. human-like).

#### Affective Organizational Commitment

Affective organizational commitment was measured by using the affective subscale of Meyer and Allen’s instrument (1991). In particular, the subscale was constituted by eight items (e.g., “I really feel as if this organization’s problems are my own”) valuable on a 5-point Likert scale (1 = *not at all*; 5 = *extremely*). Following the preliminary analysis conducted on the scale, we removed one item (i.e., item 4, “I think I could become as attached to another organization as I am to this one”) because it worsened the scale reliability and the factor solution (for more details, see Supplementary Materials section). Thus, the final score of affective commitment was computed as the average of seven items (α = .82).

### Results

#### Introductory Analyses

Overall, affective organizational commitment (*M* = 2.63, *SD* = 0.75) negatively correlated with both objectifying job features (*M* = 3.42, *SD* = 0.82), *r* = -.46, *p* < .001, and self-objectification (*M* = -0.53, *SD* = 1.48), *r* = -.43, *p* < .001. Furthermore, self-objectification was positively associated with workers’ perception of their activity as objectifying, *r* = .57, *p* < .001.

To explore the relationships between our variables by job role we conducted further correlation analyses for separated groups. Results showed that affective organizational commitment negatively correlated with both objectifying job features (*r* = -.44, *p* < .001 for salespeople; *r* = -.32, *p* = .025 for cashiers; *r* = -.64, *p* = .036 for managers) and self-objectification (*r* = -.43, *p* < .001 for salespeople; *r* = -.31, *p* = .029 for cashiers; *r* = -.61, *p* = .046 for managers) for all the job roles. Instead, the self-perception as object-like was positively associated with objectifying job features only for salespeople (*r* = .56, *p* < .001) and cashiers (*r* = .47, *p* = .001). The association between self-objectification and objectifying job features was not significant for managers (*r* = .46, *p* = .151).

Table 2 presents means and standard deviations for all the variables considered in the study by job role. As shown in Table 2, cashiers perceived their activity as more objectifying than salespeople, *t*(129) = 3.36, *p* = .001, 95% CI [0.18, 0.70], and managers, *t*(59) = 7.98, *p* < .001, 95% CI [1.26, 2.11]. Further, managers perceived their job as less objectifying than salespeople, *t*(23.26) = -8.92, *p* < .001, 95% CI [-1.54, -0.96].

**Table 2:** Means and Standard Deviations (in Parentheses) for Each Variable by Job Role

	Job role		
Variable	Salespeople	Cashiers	Managers
Objectifying job features	3.36_a_ (0.75)	3.80_b_ (0.68)	2.11_c_ (0.37)
Self-objectification	-0.41_a_ (1.30)	-0.38_a_ (1.61)	-2.13_b_ (1.20)
Affective organizational commitment	2.63_a_ (0.74)	2.50_a_ (0.72)	3.23_b_ (0.66)

*Note*. Values with different subscripts on the same row are significantly different at *p* ≤ .05.

In addition, as reported in Table 2, salespeople and cashiers reported higher self-objectification than managers, *t*(90) = 4.15, *p* < .001, 95% CI [0.90, 2.55] for salespeople; *t*(59) = 3.43, *p* = .001, 95% CI [0.73, 2.79] for cashiers. Moreover, self-objectification of salespeople and cashiers did not significantly differ, *t*(129) = -0.15, *p* = .881. Thus, while salespeople and cashiers reported similar ratings of self-objectification, managers described themselves as less similar to instrument-related words than the other two categories of workers.

Finally, managers reported more affective organizational commitment than cashiers, *t*(59) = 3.07, *p* = .003, 95% CI [0.25, 1.20], and salespeople, *t*(90) = 2.51, *p* = .014, 95% CI [0.12, 1.06]. Further, affective organizational commitment reported by cashiers and salespeople did not significantly differ, *t*(129) = -1.00, *p* = .320.

#### Main Analyses

To investigate the relationships between job role, perceived objectifying job features, self-objectification, and affective organizational commitment, we tested a double mediation model in which job role was considered the predictor variable, perception of the work activity as objectifying was the first-level mediator, self-objectification was the second-level mediator, and affective organizational commitment was the outcome variable. The double mediation hypothesis was tested using Hayes’ (2013) PROCESS macro (Model 6) and the bootstrapping method (5,000 resamples). Since the independent variable was multicategorical, by following the recommendations of Hayes and Preacher (2014), we used indicator coding. The role of the manager was coded as the reference group and was compared to the role of salesperson (D1) and cashier (D2) separately.

**Figure 1:**
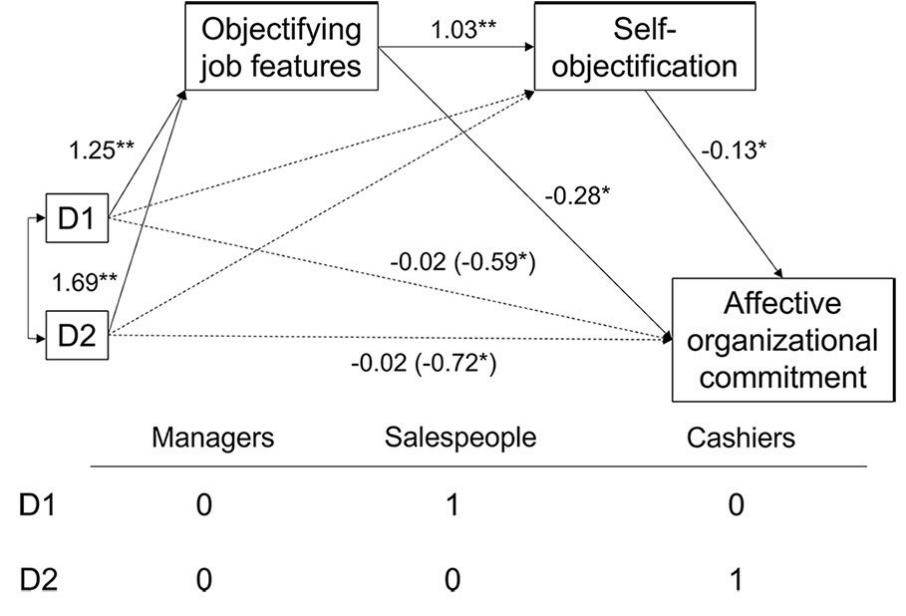
Unstandardized Regression Coefficients From the Tested Double Mediation Model *Note*. Values in brackets refer to the total effect of the independent variables. *p < .05. **p < .001.

As show in Figure 1, the effects of managers vs. salespeople (D1) and of managers vs. cashiers (D2) on the perception of objectifying job features were significant, *b* = 1.25, *SE* = 0.23, *t*(139) = 5.51, *p* < .001 and *b* = 1.69, *SE* = 0.23, *t*(139) = 7.18, *p* < .001, respectively, indicating that salespeople and cashiers (vs. managers) reported a higher perception of their activity as objectifying. In turn, perceived objectifying job features were positively related to self-objectification, *b* = 1.03, *SE* = 0.15, *t*(138) = 7.05, *p* < .001. Finally, higher levels of self-objectification were significantly related to less affective organizational commitment, *b* = -0.13, *SE* = 0.05, *t*(137) = -2.79, *p* = .006. As a first support to our double mediation hypothesis, the direct effects of both D1 and D2 on affective organizational commitment were not significant, *b* = -0.02, *SE* = 0.23, *t*(137) = -0.07, *p* = .942 for D1 and *b* = -0.02, *SE* = 0.26, *t*(137) = -0.08, *p* = .939 for D2. Importantly, the examination of the confidence intervals of the indirect effects showed that the indirect effect of managers vs. salespeople (D1) and managers vs. cashiers (D2) on affective organizational commitment via perceived objectifying job features and self-objectification were significant, the point estimate was -0.16, and the 95% CI was [-0.28, -0.05] for D1; the point estimate was -0.22, and the 95% CI was [-0.38, -0.07] for D2, supporting a double mediation model. Thus, there was support for both our hypotheses.

## Discussion

The purpose of this study was to expand research on workplace dehumanization by exploring the associations between objectifying features related to specific job roles, self-objectification, and commitment towards the organization. We assumed that, especially among workers with a low-status job role, the perception of objectifying job features would be positively related to workers’ tendency to objectify themselves. In turn, this perception would be associated with a decrease in affective organizational commitment. The double mediation analysis, as reported in the present study, supported both our expectations. In the supermarket sector, salespeople and cashiers (vs. managers) reported an increased perception of their work activity as objectifying, which in turn heightened levels of workers’ self-objectification. In turn, this self-perception was closely tied to a decreased affective commitment to the organization.

We believe that our findings extend and complement previous organizational and socio-psychological research in different ways. First, results revealed that workers with low-status job roles (i.e., cashiers and salespeople) self-objectified themselves more than workers in a high-status position (i.e., managers). These findings are in line with several studies (e.g., Baldissarri et al., 2017a, 2017b; Loughnan et al., 2017; Valtorta et al., 2019b), according to which low-status occupations characterized by repetitive movements, fragmented activities, and other direction led to an objectified view of the worker. Here we provided a step forward to the analysis of workplace dehumanization by examining self-dehumanization and expanding previous research in a real work context.

Regarding affective organizational commitment, we found that cashiers reported less commitment towards the organization than managers, but not than salespeople. In a sample of schoolteachers, Feather and Rauter (2004) showed that permanently employed workers who had secure jobs reported stronger affective and emotional commitment to their school than contract teachers, who also reported higher feelings of insecurity, perceptions of little influence or control over their role-related duties. Although we did not collect data on contracts and job insecurity in our research, it is plausible to think that workers with low-status job roles reported lower levels of affective organizational commitment because of these potential negative effects deriving from their position within the organization. Literature suggests indeed that perceptions of job insecurity might have detrimental consequences for low-status employee attitudes (Rosenblatt et al., 1999), such as an increase in job dissatisfaction (Davy et al., 1997), an increase in negative health outcomes (Hellgren & Sverke, 2003; Mohren et al., 2003), and higher reports of psychological distress (Dekker & Schaufeli, 1995; Probst, 2000). Crucially, workers with low job security perceptions are more likely to engage in work withdrawal behavior (O’Quin & LoTempio, 1998) and report lower organizational commitment (Preuss & Lautsch, 2002). Our results seem to complement this literature by providing further evidence of the negative association between low-status job roles and attachment to the organization.

Furthermore, we found a relationship between objectifying work features and self-objectification. This association is in line with the abovementioned studies; however, no previous research has assessed this link taking into account the job roles within an existing work setting. Thus, through the present field study, not only did we confirm the relation between workers’ perceptions of their activity as objectifying and self-dehumanization, but we also demonstrated that, in the work setting considered in the current research, this correlation seems to exist only for workers with low-status job roles. In this regard, it is important to note that there might be other factors at play here, which could explain why some individuals perceived their job to be repetitive and lacking autonomy. Indeed, work design literature highlights managers’ role in designing jobs (e.g., Parker et al., 2019; Parker et al., 2017), meaning that managers have the control to design stimulating, engaging jobs or dull, repetitive jobs. Therefore, while one supermarket worker in one company might be relegated to the tills for an entire day, in another company, it might be that employees are rotated around different tasks, such as restocking, serving on the tills, taking a customer service role, or ordering new stock. It is plausible that employees in the latter jobs are likely to feel that their work features are less objectifying as they are more varied and thus experienced lower levels of self-objectification.

To date, several studies (e.g., Andrighetto et al., 2018; Baldissarri et al., 2019) have reported that working self-objectification is positively associated with the key dimensions of job burnout (i.e., exhaustion and cynicism), conforming behaviors, and a decrease in belief in personal free will. Our findings provide support for the idea that the self-perception as object-like also has a negative association with workers’ affective organizational commitment by therefore impacting on both individuals’ well-being and organizational efficiency. In this respect, our results might provide an interesting integrated perspective between social-psychosocial and organizational constructs. Indeed, by showing the relationship between working dehumanization and affective organizational commitment, our research is the first to investigate the link between workers’ self-perceptions and attitudes towards the organization within a real workplace environment. Research efforts on organizational commitment have, so far, mainly focused on organizational aspects, such as turnover intensions and job performance. The present study extends these research efforts by focusing on the concept of working objectification, a somewhat neglected construct in organizational psychology and management literature, even if it is described as a frequent and common experience by workers (e.g., Bell & Khoury, 2016).

Finally, and importantly, our findings can be particularly relevant for companies. Several studies have conceptualized and tested the relationship between employees' engagement and affective organizational commitment (see Jena et al., 2017). For example, Albrecht and Andreetta (2011) found that when employees perceive that their managers have an empowering style of leadership, they feel empowered. Such feelings of empowerment will lead employees to feel engaged and also lead to feelings of belongingness to their organization. Furthermore, Schaufeli and Salanova (2007) pointed out that with the enhancement of employees' engagement, organizational commitment gets heightened. Crucially, the job demands-resources model—the most popular framework in occupational health psychology to investigate the relationships between job characteristics and employees' well-being—explains that engagement at work is much more effective than job demand in predicting organizational commitment (Hakanen et al., 2008). Our research adds a tile to this picture by demonstrating the link between working objectification and organizational commitment. Implementing labor policies that prevent the rise of self-objectification and its relative consequences in the workplace may improve workers’ psychological state and provide broader benefits for organizations and their efficiency. In addition, managers and organizations may redesign work to make it less objectifying and more stimulating and engaging, leading to organizational commitment, better well-being, and performance (for a review of work design interventions, see Knight & Parker, 2021). Related to this point, our findings can be considered a relevant starting point for future studies and interventions in the field of working motivations. Several authors (e.g., Amdan et al., 2016; Mowday et al., 1979; Wong et al., 1999) stated that motivation functions as a significant predictor of organizational commitment. With reference to the present research, extrinsic motivation—the motivation to work primarily in response to something apart from the work itself, such as reward, recognition, and benefits—may play an important role in compensating the lack of affective commitment provoked by the processes of objectification.

### Limitations and Future Directions

It is important to acknowledge that our study has some limitations that may restrict its generalizability. Although the associations we observed among variables are consistent with previous findings, the correlational nature of the current data does not allow us to draw any causal inferences. It is likely indeed that the relationships between some of our constructs are bidirectional and dynamic. Longitudinal research would be an important next step towards determining the direction of these paths.

A second important limitation concerns the (explicit) measure that we employed in our study. In particular, it is noteworthy that the mean ratings of self-objectification were negative for all the considered job roles, indicating a weak association of the workers’ perceptions with the instrument-related words. However, it should be noted that our measure assessed this association using a self-report scale, which may have been affected by the participants’ desirability concerns (e.g., Crowne & Marlowe, 1964; Nederhof, 1985). Greater associations with dehumanizing metaphors may emerge in studies using a subtler measure of dehumanization and implicit techniques, which are less susceptible to motivated responding (Gawronski & Bodenhausen, 2006). In this regard, it is important to note that the mean scores of affective commitment reported especially by salespeople and cashiers were low, and this result may be due to some work-related aspects that we did not consider in our research (e.g., number of working hours a week, number of years working within the supermarket; see, for example, Sanders et al., 2011). Further studies are needed to corroborate our findings and the relationship between workplace dehumanization and commitment towards the organization.

In addition, although the power of our sample size gives us confidence in the robustness of our results, we acknowledge that only 8% (i.e., *n* = 11) of participants were workers employed as managers in the current research. Our sample describes the reality of the considered work context (i.e., supermarket). However, we reason that a more exhaustive picture will be obtained through future studies that will replicate and expand our findings with a more balanced sample, in which all the different job roles are sufficiently and equally represented.

Furthermore, in this study, we focused only on affective organizational commitment as outcome of working objectification. However, several investigations showed that dehumanizing perceptions in the work domain are positively related to workers’ turnover intentions and negatively associated with workers’ satisfaction (e.g., Bell & Khoury, 2016; Caesens et al., 2017). By expanding these findings, future research should analyze whether objectifying job features and self-objectification can also be associated with workers’ voluntary turnover and job satisfaction.

Finally, our research focused on working objectification in a specific work setting. Nowadays, a number of jobs (e.g., call center operators; see Baldissarri et al., 2014; Pierantoni et al., 2007) are characterized by fast rhythms of work and severe forms of performance control, features that somewhat recall the Nussbaum’s facets of objectification. Thus, we believe it would be interesting to replicate and extend these findings to different work contexts.

### Conclusion

The present study contributes to the understanding of the process of workplace dehumanization by showing that a change in specific objectifying job features might lead to a change in self-objectification and affective organizational commitment. Considering that work is one of the central aspects of human life, understanding the conditions under which work becomes a source of dehumanization and their potential consequences in terms of identification with and involvement in a particular organization is an interesting task for scholars. We hope that our findings and future investigations encourage social-psychological and organizational research to join efforts in order to increase the comprehension of the impact of dehumanization on workers’ identity and well-being.

## Supplementary Materials

The supplementary materials provided are the dataset, scales, and measures used in the research and can be accessed in the Index of Supplementary Materials below).



ValtortaR. R.
MonaciM. G.
 (2023). When workers feel like objects: A field study on self-objectification and affective organizational commitment
[Dataset, scales, measures]. PsychOpen. https://osf.io/s7dra/
10.5964/ejop.5549PMC1010305937063690

## Data Availability

Data is freely available at Supplementary Materials.
